# Bovine origin *Staphylococcus aureus*: A new zoonotic agent?

**DOI:** 10.14202/vetworld.2017.1275-1280

**Published:** 2017-10-26

**Authors:** Relangi Tulasi Rao, Kannan Jayakumar, Pavitra Kumar

**Affiliations:** 1 Department of Animal Behaviour & Physiology, School of Biological Sciences, Madurai Kamaraj University, Madurai – 625 021, Tamil Nadu, India; 2 Vascular Biology Laboratory, AU-KBC Research Centre, Anna University, Chennai – 600 044, Tamil Nadu, India

**Keywords:** chicken embryo model, *Staphylococcus aureus*, virulence, zoonotic agent

## Abstract

**Aim:**

The study aimed to assess the nature of animal origin *Staphylococcus aureus* strains. The study has zoonotic importance and aimed to compare virulence between two different hosts, i.e., bovine and ovine origin.

**Materials and Methods:**

Conventional polymerase chain reaction-based methods used for the characterization of *S. aureus* strains and chick embryo model employed for the assessment of virulence capacity of strains. All statistical tests carried on R program, version 3.0.4.

**Results:**

After initial screening and molecular characterization of the prevalence of *S. aureus* found to be 42.62% in bovine origin samples and 28.35% among ovine origin samples. Meanwhile, the methicillin-resistant *S. aureus* prevalence is found to be meager in both the hosts. Among the samples, only 6.8% isolates tested positive for methicillin resistance. The biofilm formation quantified and the variation compared among the host. A Welch two-sample *t*-test found to be statistically significant, t=2.3179, df=28.103, and p=0.02795. Chicken embryo model found effective to test the pathogenicity of the strains.

**Conclusion:**

The study helped to conclude healthy bovines can act as *S. aureus* reservoirs. Bovine origin *S. aureus* strains are more virulent than ovine origin strains. Bovine origin strains have high probability to become zoonotic pathogen. Further, gene knock out studies may be conducted to conclude zoonocity of the bovine origin strains.

## Introduction

*Staphylococcus aureus* is a major opportunistic pathogen responsible for wide range of infections. The ability of *S. aureus* to cause diseases in humans and animals during immune compromised times has been attributed to its capacity to produce a variety of virulence factors [1]. The epidemiological circumstances changed mainly with the advent and spread of community-acquired methicillin-resistant *S. aureus* (CA-MRSA) strains, appearing in individuals without healthcare-associated risk factors and also in animals. Nevertheless, even where household animals and horses were known to be colonized by MRSA strains, animal-to-human (and *vice versa*) transmission marked rare evolutionary accidents rather than being the rule. In the new millennium, a novel MRSA lineage was born: Sequence type ST398 MRSA can colonize a broad spectrum of animals and has been shown to be transmitted to humans, raising the question of the advent of a new emerging zoonotic agent [2].

India is primarily based on agriculture, and most of the rural population in India actively depend on livestock for their economic growth and sustainability. In such situation, there is a significant probability for a spread of zoonotic *S. aureus*. In the dawn of this century, the evolution of multidrug resistance (MDR) *S. aureus* strains gain the attention of bacteriologists and molecular biologists. The epidemiological situations differed from the previous ages with the advent and spread of CA-MRSA strains, appearing in individuals without healthcare-associated risk factors and also in animals [2].

In this context, this study has been designed to understand the nature of *S. aureus* strains of animal origin in Madurai district, Tamil Nadu. The study primarily is focused on the characterization of animal origin strains and virulence capacity variations in strains isolated from bovine and ovine hosts. In this context, this study has been designed to understand the nature of *S. aureus* strains in Madurai district and Tamil Nadu. The study primarily is focused on the characterization of zoonotic strains and virulence capacity variations in zoonotic strains isolated from bovine and ovine hosts.

## Materials and Methods

### Ethics approval

The authors declare that the research was conducted with prior approval from Institutional Biosafety and Ethical Committee (IBEC) of the School of Biological Sciences, Madurai Kamaraj University(Dated 14 Nov 2014).

### Sample collection

Nasal swabs collected from healthy adult animals include cows (Bovines) and sheep (Ovine) with sterile cotton swabs (HiMedia Sterile culture collecting device) in Madurai district during October 2015-March 2016. Collected samples transported to the laboratory in Cary-Blair transport media. The samples processed on the day of sample collection itself to ensure the maximum recovery of bacteria. Samples not more than five were collected to provide heterogeneity from any herd or farm.

### Initial screening for *S. aureus*

The nasal swabs collected initially spread on Mannitol salt agar (MSA) and Baird-Parker Agar, a selective media for *S. aureus*. Further Gram staining performed for positive isolates on agar plates. Plates incubated at 37°C for 24-48 h.

### Multiplex polymerase chain reaction (PCR) amplification

For molecular characterization, PCR-based methods were used. DNA isolation performed with the aid of Favorogen Tissue DNA extraction kit, Taiwan. DNA extracted according to the manufacturer’s protocol. Lysostaphin (Sigma, USA) is used for better lysis of the cell walls as per manufacturer’s recommendation. The PCR conditions and primers used as described in the previous literature. Two different multiplex PCR reactions have been carried out based on standard methods [3-5] with little modifications, to confirm *S. aureus* at species level and methicillin resistance. One multiplex PCR reactions with the *Staphylococcus-*specific *16s rRNA*, *nuc*, and *sdrE* and another set comprised *Staphylococcus*-specific *16s rRNA, mecA*, and *sdrE*. PCR products were resolved on agarose (1% w/v) gel electrophoresis.

### Virulence analysis carried out with following experiments

#### Assessment of biofilm formation

Biofilm quantification was carried out according to the protocol of O’Toole [6]. Absorbance quantified using microtiter plate reader (Spectramax M2, Molecular Devices, USA). Cells were grown on 96-well costar plates and incubated at 37°C for 24 h for visualization of biofilm development. After the incubation, the wells were washed with sterile phosphate-buffered saline and stained with acridine orange (0.1% w/v), and the biofilms were imaged using high-content screening (HCS) using Operetta imaging system (Perkin Elmer, Germany). The Z-stack analysis performed using Harmony software 2.0.1.

### In vivo estimation of virulence - chicken embryo infection model

The estimation of the virulence capacity of strain was performed using chicken embryo as a model. The experiment conducted for few selected strains, whereas MRSA- MTCC1430 served as the positive control and saline (0.7% NaCl) as a negative control. The experiment carried according to the protocol developed earlier with the hatching eggs obtained from commercial hatcheries and ensured feed does not contain any antibiotic [7]. Briefly, the eggs were incubated at 37°C and a relative humidity of 50-60% in a laboratory incubator. On the 10^th^ day of development, the eggs candled and dead and unfertilized embryos removed. A small hole was aseptically drilled in the eggshell using a needle, and a suspension (50 µl, ~ 10^6^ colony-forming units [CFUs]) of the *S. aureus* strain being tested was inoculated intraallantoically, and 20 eggs per group challenged with bacterial strain. The liver damage further confirmed by histopathological analysis.

### Statistical analyses

All statistical analyses executed on R program (Welch two sample t-test), version 3.0.4 on local Linux machine.

## Results

**Figure-1:**
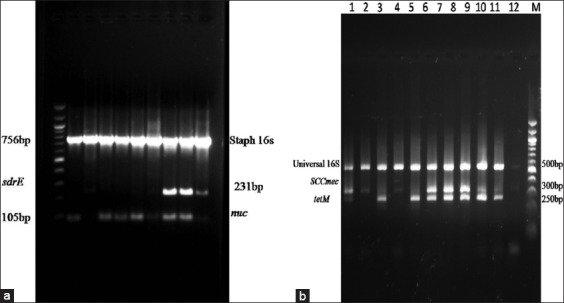
Multiplex polymerase chain reaction (PCR) analysis of different combinations of genes. (a) A combination with *Staphylococcus-*specific *16s rRNA*, *sdrE*, and *nuc* genes, L1- 100bp ladder, (b) multiplex PCR analysis of antibiotic resistance genes zoonotic *Staphylococcus aureus* strains L1- methicillin-resistant *S. aureus* MTCC1430, L2- 11 – isolates L12 –NTC L13 – 100 bp ladder. Here, Universal *16s rRNA* primer used as positive control.

Samples collected in Madurai district from adult healthy animals only. The sampling details found in Figure-1. A total of 128 samples collected in Madurai district mostly with the rural backdrop.

### Initial screening for *S. aureus*

The nasal swabs collected were initially spread on MSA and Baird-Parker Agar, a selective media for *S. aureus*. Further, Gram staining performed for positive isolates on agar plates. After incubation at 37°C for 24 h, the agar plates observed for the growth of bacterial colonies. The colonies with yellow to golden yellow color on MSA plates and black colonies on Baird-Parker agar plates selected for further screening. 30 samples from the bovine origin and 20 samples from ovine origin were positive on both selective media agar plates. Gram staining performed for further confirmation. False-positive samples eliminated with Gram staining. After Gram staining, 32 samples from bovine origin exhibited spherical-shaped morphology and Gram-positive and 25 samples from ovine origin positive for *S. aureus* morphology.

### Molecular characterization

Two multiplex PCR reactions were carried out for further confirmation. Multiplex PCR performed with reported species-specific primers. In this study, one multiplex PCR carried out with *Staphylococcus-*specific *16s rRNA, nuc*, and *sdrE* gene primers to confirm isolated strain as *S. aureus* at molecular level. Another multiplex carried out with a little modification to assess the strain’s methicillin resistance at the molecular level. In this set, *nuc* gene primer replaced with *mecA* primer. Figure-1a and b illustrated the multiplex PCR results. After PCR confirmation, 26 isolates from the bovine origin and 19 samples from ovine origin confirmed as *S. aureus*. Among these isolates, 10 isolates confirmed as MRSA based on the presence of *mecA* gene in their genome.

### Prevalence analysis

**Figure-2:**
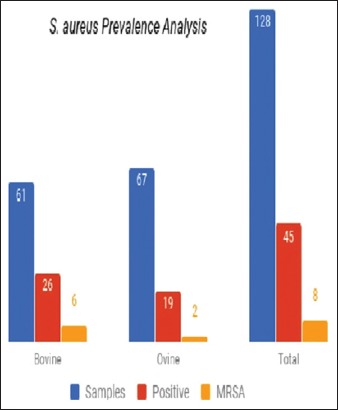
Prevalence analysis. Bar diagram is representing the prevalence rate of *Staphylococcus aureus* and methicillin-resistant *S. aureus* in bovine and ovine samples.

After molecular confirmation, the prevalence rate of *S. aureus* in the study population was analyzed. It has been observed that the prevalence rate in bovine origin samples was around 42.62%, while it was low in the case of ovine origin samples with 28.35% only. The prevalence rates can be seen in a bar diagram with MRSA rates as well (Figure-2). The prevalence rate of MRSA found to be very meager to an extent of 6.8%.

### Virulence analysis

Different experiments including *in vivo* virulence analysis performed to estimate the virulence capacity of the positive *S. aureus* isolates of this study and to compare the host-based virulence assessment.

### Biofilm formation capacity

Biofilm formation is a potential virulence factor in *S. aureus* isolated from clinical mastitis [8]. Hence, to have an understanding of the disease establishment by *S. aureus* strains from healthy animals, the biofilm formation assessment was carried out. In Table-1, the mean and standard deviation values of biofilm of each strain of this study enlisted. The results showed that the strains were weak to high capacity of biofilm formation. A parametric statistic test, Welch two sample *t*-test used to detect the variation of biofilm formation among the host-based specificity. This test found to be statistically significant, t=2.3179, df=28.103, and p=0.02795. This result suggests bovine origin *S. aureus* strains has the better capacity of biofilm formation than the ovine origin strains.

**Table-1:** Biofilm quantification of *S. aureus* strains.

Biofilm quantification			

Bovine origin strains		Ovine origin strains	
	
Sample ID	Mean±SD	Sample ID	Mean±SD
A1	0.0384±0.007635	C1	0.0402±0.011212
A2	0.0386±0.028086	C2	0.0734±0.01491
A3	0.0434±0.009529	C9	0.0478±0.012988
A5	0.1228±0.0561	C12	0.0472±0.008438
A6	0.0694±0.038188	C13	0.0768±0.018089
A7	0.1254±0.043362	C15	0.144±0.06804
A8	0.1186±0.023158	C16	0.1026±0.020888
A9	0.0322±0.0113	C18	0.0884±0.02446
A10	0.0654±0.009864	C19	0.0636±0.019008
A12	0.0314±0.00929	C22	0.0606±0.030648
A13	0.0796±0.007301	C25	0.052±0.007106
A15	0.0476±0.0177	C29	0.0616±0.015192
A19	0.0506±0.010262	C30	0.0928±0.016634
A20	0.0412±0.02955	C31	0.0434±0.013975
A21	0.0368±0.017635	C32	0.049±0.011895
A22	0.055±0.015427	AG7	0.0378±0.012637
A24	0.0508±0.011389	AG9	0.0434±0.023533
A25	0.2442±0.010918	CS5	0.0382±0.008438
A27	0.0432±0.009121	CS7	0.0638±0.008468
O1	0.0842±0.033641		
B1	0.18625±0.017443		
B2	0.176±0.049221		
B3	0.30375±0.082553		
B8	0.255±0.02735		

*S. aureus=Staphylococcus aureus*, SD=Standard deviation

### Visualization of biofilm

**Figure-3:**
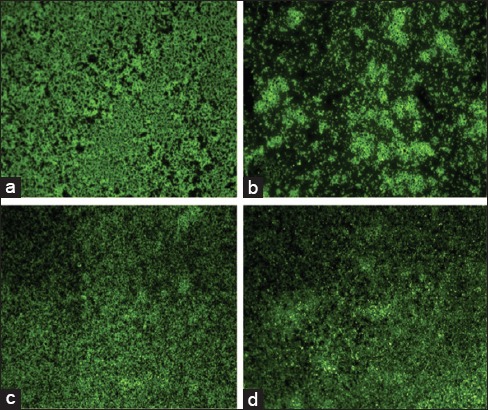
Biofilm formation of different strains of *Staphylococcus aureus*, (a) biofilm formed by methicillin-resistant *S. aureus* (MRSA) MTCC 1430, (b) moderate biofilm formed by ovine origin *S. aureus*, (c) biofilm formation of bovine origin MRSA, and (d) biofilm formation of bovine origin *S. aureus*.

Biofilms imaged by HCS using Operetta imaging system. The images showed the difference in biofilm formation among the different strains. Figure-3 illustrated the biofilm formation of different strains.

### Chicken embryo assay

**Figure-4:**
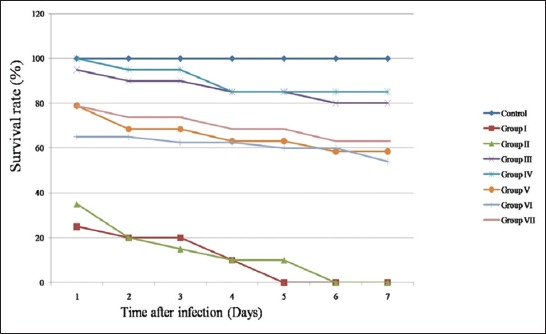
Survival analysis curve. The curves are representing the survival rates of chicken embryos from day 1 to day 7 after challenging with *Staphylococcus aureus*. Control: 0.7% NaCl, Group I - methicillin-resistant *S. aureus*, Group II - human origin *S. aureus*, Group - III and IV - ovine origin *S. aureus* (Sample ID C15 and C30), and Group V – VII – bovine origin *S. aureus* strains (Sample IDs- A25, B1, and B2, respectively).

Since the biofilm formation is the key criteria for initial adherence and disease establishment, and again, it is also acts as a virulence factor in disease establishment [8] the strains which are capable of more biofilm (Table-1) along with which positive for toxic shock syndrome toxin gene *(tsst-1*, data not shown) selected for *in vivo* analysis. The mean survival rates noted till 7^th^ day after the commencement of the experiment. On the 7^th^ day, liver dissected from euthanized embryos and tested for the confirmation of pathogen with counted conventional serial dilution method. The results showed the presence of *S. aureus* in liver samples and evident for bacterial colonization. CFU counted conventionally. MANOVA analysis of CFUs and liver weight for different groups with respect to control yielded a significant difference among groups after challenging with pathogen. Results from this MANOVA demonstrated a significant multivariate effect for the relationship, Wilkin’s Λ (7, 21) = 0.011, p<0.0001. The survival analysis of embryos also performed till the end of the experiment and results illustrated in Figure-4.

### Histopathological studies

**Figure-5:**
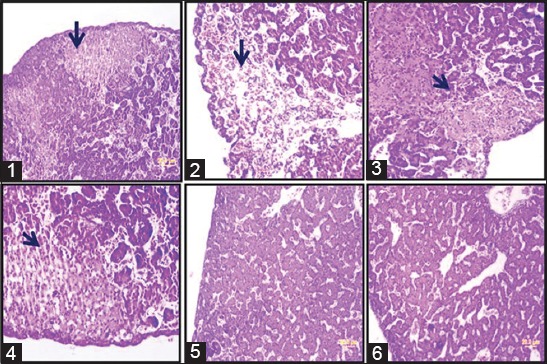
Histopathological analysis. Histopathology analysis of liver cross-sections of chick embryos showed liver fibrosis. Fibrosis was marked with an arrow mark. (1) Methicillin-resistant *S. aureus* (Group I), (2) human origin (Group II), (3) bovine origin (Group V), (4) bovine origin (Group VI), (5) ovine origin, and (6) control. In ovine origin and control, no fibrosis observed.

Histopathological studies carried to check liver damage by pathogen (Figure-5). As expected with respect to control fibrosis noticed in the liver samples challenged with pathogen but no fibrosis observed liver samples which challenged with ovine origin strains.

## Discussion

*S. aureus* being an opportunistic pathogen and MDR superbugs have been reported recently, and hence, the scientific community interested in studying the evolutionary trends of this bacterium. The present study focused on the prevalence and pathogen potentiality of zoonotic origin strains. Nasal swabs used as samples since it is the prescribed sampling method [9]. After initial screening and molecular characterization of the prevalence of *S. aureus* found to be 42.62% in bovine origin samples and 28.35% among ovine origin samples. Meanwhile, the MRSA prevalence is found to be meager in both the hosts. Among the samples, only 6.8% isolates tested positive for methicillin resistance. Despite the prevalence rate seems to be very less, the hosts still acts as reservoirs. However, the reason for the prevalence of *S. aureus* found more in bovines (cows) while compared to that of ovine samples is yet too established.

The majority of the prevailing reports focused mainly on human origin and bovine mastitis *S. aureus* [10,11]. MRSA prevalence results can be comparable with Haran *et al*.’s [12], wherein they are reported only as 4% of MRSA in herd animals. In India, Kumar *et al*. [13] also reported that prevalence of 13.1% MRSA in Sahiwal cattle. In contrast to animal sample, milk carries less *S. aureus* as it was noticed in the reports of Fagundes *et al*. [14] and Thaker *et al*. [15] which revealed the presence as 7.3% and 6.25%, respectively. Increasing number of samples may alter the prevalence rate and helps to obtain the approximate prevalence in the study population.

The prevalence of any pathogen and its epidemiological importance can be confirmed only after confirming the strain with the aid of molecular tools only. Molecular characterization of *S. aureus* performed with established protocols in accordance to the previous studies. In this study, *Staphylococcus-*specific *16S rRNA, nuc*, and *sdrE* gene primers used to confirm the bacterial isolate as *S. aureus*. Duran *et al*. [16] developed multiplex PCR using *16S rDNA, mecA*, and *femA* gene primers to characterize *S. aureus*. Meanwhile, the widely used gene primers were used to characterize MRSA strain such as *nuc* and *mecA* in the study [3-5].

### Virulence capacity assessment

For any opportunistic pathogen, it is mandatory to assess its virulence capacity. The virulence capacity of a strain can be determined with several molecular, *in vitro* and *in vivo* studies. *S. aureus* considered as one of the strong biofilm forming bacteria which in turn helps to gain resistance against antimicrobials [17]. Biofilm formation regarded as a potential virulence factor in *S. aureus* [8]. Hence, quantifying the biofilm gives an overview about the strains pathogenicity. Quantification of biofilm revealed that the strains of the bovine origin seem to be successful biofilm formers than the ovine origin strains. The reason behind this is not yet established. Lee *et al*. [18] assessed the biofilm-producing ability of *S. aureus* isolates from Brazilian dairy farms. They reported 45% of strains isolated from a dairy farm, while comparison of biofilm-forming capacity of MRSA strains is seemingly found less. MRSA strains form biofilm at a higher rate as reported by Cha *et al*. [19].

Chicken embryo model used to estimate the virulence potential of some selected isolates. Even though mice are widely used as an animal model to study pathogenesis, chicken embryo model is economical and reliable. Mice are used for pathogenesis and infection studies. Even though mice are easy to handle and breed but expensive to maintain. However, whether mice are an appropriate model for pathogen studies have often been discussed based on the assumption that mice are not natural hosts [20]. *Caenorhabditis elegans*, *Drosophila melanogaster*, and *Danio rerio* (Zebrafish) models employed to study pathogenesis of bacterial pathogen but their temperature maximum (≤30°C), which is fairly distant from the temperature of a human infection and activation of virulence gene expression (37°C), makes them less suitable for this study [21]. Based on Polakowska *et al*. [7] findings wherein chicken embryo model used to estimate the virulence capacity of isolates. The colonization found to be less in ovine origin strains and moderate in bovine origin strains. These results are in accordance with survival rate of embryos after infection reported by Polakowska *et al*. [7]. The survival rate varies from 60% to 80% in the bovine origin strain-infected group. As expected, MRSA type strain exhibited more virulence (at the end of experiment survival rate 0%). Histopathological studies also supporting the earlier results and but no fibrosis seen in ovine origin strain challenged group; this may be due to host immune resistance. To our search knowledge, there are no reports available on histopathological-related studies of chick embryo liver samples treated with *S. aureus*.

## Conclusion

Even though the study carried with moderate number of samples for comparison studies but acts as a preliminary study base for large-scale studies. Even the prevalence rate of *S. aureus* seems to be less in the study population but cautious enough to conclude the occurrence of *S. aureus* in healthy animals. The virulence assessment studies are evident for the bovine origin *S. aureus* strains are more virulent when compared with ovine origin. This study clearly shows that bovines can act as reservoirs for virulent *S. aureus*. Further, this bovine origin *S. aureus* may be a new zoonotic bacterial pathogen.

## Authors’ Contributions

RTR, KJ, designed experiments. RTR and PK executed experiments. RTR, KJ, and PK prepared and corrected manuscript. All authors read and approved the final manuscript.
